# Preparation and Characterization of Patch Loaded with Clarithromycin Nanovesicles for Transdermal Drug Delivery

**DOI:** 10.3390/jfb14020057

**Published:** 2023-01-19

**Authors:** Ahlam Zaid Alkilani, Batool Musleh, Rania Hamed, Lubna Swellmeen, Haneen A. Basheer

**Affiliations:** 1 Department of Pharmacy, Faculty of Pharmacy, Zarqa University, Zarqa 13110, Jordan; 2 Department of Pharmacy, Faculty of Pharmacy, Al-Zaytoonah University of Jordan, Amman 11733, Jordan; 3 Department of Pharmaceutical Chemistry, Faculty of Pharmaceutical Sciences, Hashemite University, Zarqa 13133, Jordan

**Keywords:** Clarithromycin, niosomes, transdermal, drug delivery, patch

## Abstract

Clarithromycin (CLR), categorized as a Biopharmaceutical Classification System class II drug, has several gastrointestinal tract side effects and an extremely unpalatable bitter taste. The current study aimed to design transdermal patch-embedded CLR niosomes to overcome the aforementioned CLR-related challenges. Various niosomal formulations were successfully fabricated and characterized for their morphology, size, in vitro release, and antimicrobial efficacy. Subsequently, the CLR niosomes were loaded into transdermal patches using the solvent casting method. The polydispersity index of the niosomes ranged from 0.005 to 0.360, indicating the uniformity of the niosomes. The encapsulating efficiency (EE)% varied from 12 to 86%. The optimal Chol: surfactant ratio for drug release was found to be 0.5:1. In addition, the encapsulation of CLR into niosomal nanovesicles did not reduce the antibacterial activity of the CLR. The niosomal patch had a significantly higher permeability coefficient of CLR than the conventional patch. In addition to that, a shear-thinning behavior was observed in the niosomal gels before loading them into a niosomal patch. The flux (Jss) of the niosomal patch was significantly higher than the conventional patch by more than 200 times. In conclusion, niosome-based transdermal patches could be a promising method for the transdermal drug delivery of class II drugs and drugs experiencing GIT side effects.

## 1. Introduction

The abundance of blood arteries and lymphatic vessels in the skin that are closely connected to the rest of the body provide an ideal route to transport therapeutic substances to the skin [1]. However, the skin barrier not only protects the body from harmful toxins but also severely impairs drug absorption through the skin [1]. Most drugs cannot easily traverse the skin due to the stratum corneum (SC) that restricts their penetration [1,2]. Transdermal drug delivery (TDD) has drawn a lot of attention over the past 20 years due to its many advantages over conventional drug delivery methods, including its simplicity, pre-determined doses, ease of handling, patient self-administration, and relatively simple storage requirements, whereas, the oral route of drug delivery has several disadvantages when it comes to drugs with limited solubility, permeability, and stability [2,3]. TDD also has many benefits over oral drug delivery, including avoiding first-pass metabolism, protecting sensitive drugs from the harsh gastrointestinal environment, allowing for sustained drug release, and maintaining a more uniform plasma concentration [2,3]. In order to increase the permeability of drugs across the SC, passive approaches are used to influence drug and vehicle interaction such as permeation enhancers, nanoemulsions, and vesicles [2,3,4,5]. In general, most of the passive techniques such as nanoemulsion and niosomes can be easily included into transdermal patches.

Over the years, niosomes have been studied as a promising drug delivery platform for various administration routes, including oral, dermal, and transdermal routes [6,7,8]. Niosomes have the extraordinary capacity to entrap hydrophilic, lipophilic, and amphiphilic compounds. Niosomes have advantages over liposomes, such as superior stability, low cost, simplicity in formulation, and scaling-up [9,10,11]. Transdermal patches are one of the novel pharmaceutical dosage forms for delivering drugs to the bloodstream after application to the skin [3,12]. The patches are meant to distribute drugs in a regulated, modified, and time-limited manner at a predetermined rate [12].

CLR is a prescription macrolide antibiotic [13]. It is administered orally to treat certain bacterial infections caused by specific types of bacteria, such as community-acquired pneumonia, throat infections (pharyngitis), acute sinus infections, H-pylori, and others [14,15]. In the Biopharmaceutical Classification System (BCS), CLR is categorized as a class II drug since it is mostly insoluble in water (0.33 mg/L) and exhibits a pH-dependent solubility [14,16,17]. It has several gastrointestinal tract (GIT) side effects such as diarrhea, vomiting, nausea, headache, and stomach pain [18]. CLR’s terminal half-life is 3–4 h, necessitating repeated dosage (2–3 times daily), and its bioavailability is 50% [18,19]. In addition, it has an extremely unpalatable bitter taste, which may decrease patient adherence especially in pediatrics [20]. Zhu et al. [21] reported a strong correlation between children’s adherence and the drugs’ palatability. According to retrospective studies, medicines with poor palatability have a detrimental impact on adherence, whereas those with good palatability have a positive influence [22,23]. Niosome-based patches that mediate TDD are a potential strategy for avoiding the bitterness of oral liquid dosage forms of CLR, lowering GIT-related side effects and extending CLR’s presence in the systemic circulation, thus allowing for less-frequent dosing. Furthermore, as niosomal patches are simple needle-free alternative systems, they enhance pediatrics’ adherence, which may help to reduce antibiotic resistance.

This study aimed to develop CLR-loaded niosomal nanovesicles that were incorporated into a skin patch for transdermal administration to avoid CLRs’ bitterness and prevent gastrointestinal side effects. Additionally, TDDs can decrease antibiotic resistance by lowering the dose of antibiotics administered. The entrapment effectiveness, shape and size of the vesicles, zeta potential, in vitro release, and ex vivo permeability were determined as the physicochemical features to be studied for CLR niosomal formulations. After that, CLR niosomal nanovesicles were incorporated into transdermal skin patches and characterized for their thickness, drug content, moisture content, and ex vivo permeation.

## 2. Materials and Methods

### 2.1. Materials

Clarithromycin was obtained as a gift sample from the JOSWE Pharmaceutical Company (Amman, Jordan). Phosphoric acid was purchased from RomiL™ (Cambridge, UK), while HPLC-grade acetonitrile and HPLC-grade methanol were purchased from Tedia™ (Fairfield, OH, USA). Span™ 40, Span™ 60, Span™ 80, Tween™ 60, Tween™ 80, and cholesterol (Chol) were purchased from Sigma Aldrich™ (Gillingham, Dorset, UK). Diethyl ether and methanol were purchased from Tedia™ (Fairfield, OH, USA). Cellulose dialysis membranes with a molecular weight cut-off (MWCO) of 12–14 kDa, average flat width of 28.46 mm, and average diameter of 17.5 mm were purchased from HiMedia Laboratories™ (Mumbai, Maharashtra, India). Polyethylene glycol 400 (PEG 400) was purchased from GCC Diagnostics™ (Deeside, Flintshire, UK). Hydroxypropyl methyl cellulose (HPMC) 40,000, dicetyl phosphate (DCP), and phosphate-buffered saline (PBS) tablets were purchased from Sigma Aldrich™ (Gillingham, Dorset, UK). All other chemicals used in this study were of analytical grade.

### 2.2. Saturation Solubility of CLR

At 37 °C, CLR’s saturation solubility was tested using PBS (pH 7.4) and PBS containing 20% isopropyl alcohol. In a 25 mL beaker, an excess amount of CLR was added to 10 mL of each solvent. The beakers were securely covered and shaken for 24 h at 37 °C and 100 rpm in a shaker incubator. HPLC analysis was used to assess the saturation concentration of CLR after aliquots of the solution were removed, filtered, and centrifuged at 1400 rpm for 15 min.

### 2.3. Preparation of CLR Niosomes

The ether injection method was used to prepare niosomal formulations. In brief, weighed amounts of different nonionic surfactants (Span 40, Span 60, Span 80, and Tween 80) were combined with Chol and dissolved in 16 mL of diethyl ether before being mixed with 4 mL of methanol containing a weighed amount of CLR, and then the required amount of dicetyl phosphate (DCP) was added to the mixture. The resultant solution was then slowly injected into 20 mL PBS (pH 7.4) using a syringe pump at a rate of 1 mL/min. The solution was then constantly stirred with a magnetic stirrer while the temperature was kept at 60–65 °C. Because of the temperature differences between the phases, evaporation occurred, resulting in the formation of niosomes. To choose an optimum niosome formulation, various formulations were prepared. Table 1 shows the amounts of CLR, surfactants, and Chol. The prepared niosomal dispersions were kept refrigerated at 4 °C.

### 2.4. Characterization of CLR-Loaded Niosomes

#### 2.4.1. Transmission Electron Microscopy (TEM)

The morphology of the niosomes was observed using a transmission electron microscope (TEM, FEI Morgani 268, operating voltage of 60 kV, Holland) connected to a Mega View II digital camera. One drop of the niosomes was diluted with distilled water, placed on a carbon-coated copper grid, and allowed to dry before imaging. The morphology of the niosomes was estimated by the ImageJ software.

#### 2.4.2. Particle Size (PS), Zeta Potential (ZP), and Polydispersity Index (PDI)

The PS, ZP, and PDI of the prepared formulations were assessed using a particle size analyzer (Brookhaven 90 plus, NY, USA). The internal Zetasizer software automatically presented the PDI for all of the particles examined. Each experiment was repeated three times, and the findings were provided as mean ± SD. For the ZP of the niosomes, electrophoretic light scattering (ELS) was used to determine the surface charge of the particles. At 25 °C, the niosomes were dispersed in distilled water and sonicated until a clear solution was obtained. Each experiment was carried out in triplicate, and the data were provided as mean ± SD.

#### 2.4.3. Encapsulation Efficiency (EE)

Centrifugation was used to measure the %EE of all the niosomal formulations. For this purpose, a niosomal suspension was poured into 1.5 mL Eppendorf and centrifuged for 1 h at 16,000 rpm at 4 °C using a cooling centrifuge. The supernatant was discarded, and the separated niosomes were washed with PBS before being centrifuged an additional two times under the same conditions. The amount of the entrapped drug was determined by adding 1 mL of isopropanol to 0.1 mL of the separated niosomes, which was then diluted with PBS up to 20 mL and then sonicated and centrifuged again at 14,000 rpm for 15 min at 25 °C to obtain a clear solution to be analyzed by HPLC. The EE (%) was determined using Equation (1), as follows:

(1) $$\mathrm{EE}\left(\%\right)=\frac{\mathrm{Amount}\;\mathrm{of}\;\mathrm{entrapped}\;\mathrm{drug}}{\mathrm{Total}\;\mathrm{CLR}\;\mathrm{amount}}\times 100\%$$

where the Amount of entrapped drug is the actual amount of the drug successfully encapsulated in the vesicles and the total CLR amount refers to the entire quantity of CLR utilized during preparation.

#### 2.4.4. In Vitro Release Studies

The dialysis bag method was used to test the in vitro release pattern of the niosomal suspensions. PBS was used to wash and soak the dialysis bags for 24 h. A niosomal suspension (equivalent to 14.28 mg CLR) was diluted in 2 mL PBS. The dialysis bag was then placed in 20 mL PBS containing 20% isopropanol and incubated for 24 h at 37 °C and 100 rpm. To keep the sink condition throughout the experiment, a 1 mL sample was taken at specified intervals and promptly replaced with 1 mL of fresh medium. HPLC was used to analyze the samples.

#### 2.4.5. Release Kinetics Modeling

Using LabPlot version 2.0, the drug release rates for the selected niosomal formulations (F1 and F9) were fitted to the Korsmeyer–Peppas equation (Mt = K* × t^n^) and first-order kinetics. The software was then given the task of determining the best fit line. Only fit curves with R^2^ ≥ 0.95 and the sum of squared residuals (SSD) ≤ sum of squares were considered appropriate.

#### 2.4.6. Antimicrobial Activity Testing

The agar well plate diffusion method was used to ensure the antimicrobial properties of the niosomal formulations according to the Clinical and Laboratory Standards Institute (CLSI) for bacteria and yeast testing [24]. The antibacterial activity of the CLR-loaded niosomes (F9) and CLR was assessed using inhibitory zone measurements against Gram-negative and Gram-positive organisms, including *Staphylococcus aureus* (*S. aureus*) (ATCC 6538) and *Escherichia coli* (*E. coli*) (ATCC 8739), respectively. Microbial suspensions of *S.aureus* and *E. coli* were produced in sterilized normal saline to achieve a bacterial cell concentration of 10^8^ CFU/mL (0.5 McFarland turbidity) [25]. The wells (9 mm in diameter) were prepared by using sterile cork borer, and an overnight bacterial inoculum was uniformly spread using a sterile cotton swab over a sterile Mueller–Hinton agar plate. The chosen niosomal formulations, CLR working standard solutions (12 mg/mL for *S. aureus* and 30 mg/mL for *E. coli*) as the positive controls, and (1:1) DMSO and PBS as a negative control were added to the wells and incubated for 24 h at 37 °C. After incubation, confluent bacterial growth was observed. The inhibition zone of the bacterial growth was measured in mm.

### 2.5. Fabrication of CLR-Loaded Niosomal Patches

**Figure 1:**
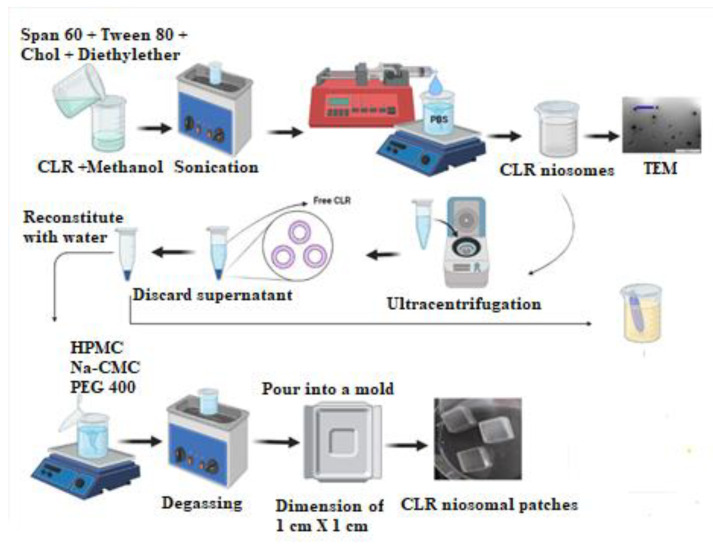
Schematic representation of the fabrication of CLR-loaded niosomal patches.

CLR-loaded niosomal patches (CLR–NP) were prepared using the solvent evaporation method as described in [26]. Different ratios of 5% *w*/*w* aqueous solution of HPMC and/or 4% *w*/*w* aqueous solution of Na-CMC were used in the preparation of the niosomal patches, and PEG400 was introduced as a plasticizer. The chosen niosomal formulation (F9) was reconstituted in 2 mL distilled water and added to the mixture of aqueous polymeric solution. After that, the mixture was thoroughly stirred using a magnetic stirrer to achieve homogeneity and sonicated for 15–20 min to remove air bubbles. The mixture was then poured into glass molds and dried at room temperature for 24 h. Table 2 shows the proportion of HPMC, Na-CMC, PEG 400, and the used niosomal formulation. Figure 1 illustrates the fabrication procedure for CLR–NP. All the prepared patches were then removed from the molds and visually examined for color, homogeneity, flexibility, brittleness, and smoothness.

### 2.6. Evaluation of Niosomal Patches’ Physicochemical Properties

#### 2.6.1. Physicochemical Evaluation of CLR-Loaded Niosomal Patches (CLR–NPs)

A digital caliber (Mitutoyo Co., Sakado, Japan) was used to measure the thickness of the CLR–NPs at three distinct positions on the patches. For each CLR–NP, the mean ± SD was recorded. Weight uniformity was determined by weighing the patches and calculating the samples’ average weights ± SD. Surface pH was determined by immersing the patches in 0.5 mL of double-distilled water for 1 h. The surface pH was then measured by placing the probe of a digital pH meter near the patch surface and allowing it to equilibrate. This experiment was carried out in three replicates, and the data were provided as mean ± SD.

#### 2.6.2. Moisture Content Determination

The prepared patches were carefully weighed and kept in a desiccator containing calcium chloride for 72 h at room temperature [27]. The patches were reweighed, and the percentage moisture content was calculated using Equation (2):

(2) $$\%\;\mathrm{Moisture}\;\mathrm{content}\;=\left(\frac{\mathrm{Initial}\;\mathrm{weight}-\mathrm{Final}\;\mathrm{weight}}{\mathrm{Final}\;\mathrm{weight}}\right)\times 100$$

#### 2.6.3. Drug Content

The CLR content in each patch was evaluated by dissolving 1.7 cm^2^ patches in 20 mL PBS at 37 °C and 100 rpm and then centrifuging them at 14,000 rpm for 15 min at 25 °C to provide a clear solution, and the samples were then quantified using HPLC. The test was carried out in triplicate, and the mean ± SD was recorded.

#### 2.6.4. ATR–FTIR

To assess any incompatibilities a Perkin Elmer UATR-II device was used for attenuated total reflectance–Fourier transform infrared spectroscopy (ATR–FTIR). Spectra were acquired in the absorbance mode, and the resolution was set to 2 cm^−1^ with 32 scans per sample. The acquired spectral data were exported in .CSV format and analyzed using Spectrograph Version 1.2.15. CLR (pure drug), Span 60, Chol, Tween 80, CLR niosomes, HPMC, Na-CMC, PEG 400, the physical mix of components, and niosomal patch (P4) spectra were all studied using ATR–FTIR.

### 2.7. Rheological Study

The viscosity of the CLR niosomal gels loaded with the niosomal formulations (F9) and the conventional CLR gel (control) was measured in triplicate at 32 °C using a cone–plate geometry (gap of 0.1 mm, cone diameter of 25 mm, and cone angle of 1°) on a controlled-stress rheometer (CSR) as described in [28]. Gel samples (CLR niosomal gels and control) weighing 0.5 g were placed onto the plate and allowed to relax for 1 min before testing. The viscosity of each gel was measured as a function of the applied shear rate, which ranged from (0.1 to 100 s^−1^).

### 2.8. In Vitro Permeation Studies

The Franz cell apparatus (PremeGear, PA, USA) was used to explore the permeation of CLR from the niosomal patches. The effective diffusion surface area was 1.767 cm^2^, and the receiving chamber volume was 12 mL. A Strat-M^®^ membrane was used without soaking or hydration [29,30]. The membrane was retained in place between the vertical diffusion cells’ donor and receptor compartments. The receptor compartment was filled with 12 mL of PBS solution containing 20% isopropanol. The receptor compartment was set at 32 °C, which was similar to the temperature of skin [31]. To simulate in vivo conditions, continuous stirring was ensured at 600 rpm. The niosomal patches with a surface area of 1.767 cm^2^ were placed in the donor compartment. To prevent evaporation, the donor compartment was coated with parafilm™. At 1, 2, 3, 4, and 24 h, 1.0 mL of each Franz cell content was removed using 1 mL syringes. To maintain the sink conditions, an identical volume of fresh PBS (pH 7.4) containing 20% isopropanol was carefully introduced to the receptor compartment, ensuring not to trap air behind the membrane. Samples were quantified for their CLR content using HPLC. The % cumulative amount of CLR that permeated through the membrane (%Q) was plotted against time (t). The slope of the linear element of the cumulative amount of CLR permeated per unit area (Q/A) vs. time plot was used to determine the steady-state flux (Jss, µg/cm^2^/h), where A is the surface area of the Franz cell opening, which was 1.767 cm^2^.

The apparent permeability (*P*) was determined using Equation (3):

(3) P=Jss/Co

where *Co* is the initial drug concentration in the donor compartment.

### 2.9. Cytotoxicity Assay

A cytotoxicity assay for the CLR niosomal patches (P4) and the blank niosomal patches was performed according to the MTT cytotoxicity assay as described in [32]. Briefly, MCF-7 cells were seeded in 96-well plates at concentrations of 5000 cells/well and cultured in the presence of either a CLR transdermal patch (P4) or a blank patch (without CLR). After 72 h of treatment, 20 μL of MTT (5 mg/mL) was added for 4 h to each well. Following this, the media in the wells were removed, 200 μL of dimethyl sulfoxide was added, and the absorbance was then measured at 560 nm.

### 2.10. Assay Method

Separation was carried out on a C18 column (4.6 mm × 15 cm), (Phenomenex, Torrance, CA, USA) using a mixture of methanol and 0.0335 M monobasic potassium phosphate (1:1), with the pH adjusted to 4.0 using phosphoric acid, and the samples were then passed through a mixed-cellulose membrane filter. All samples were filtered through 0.22 μm Minisart RC4 filters (Sartorius AG) before analysis. Then, the mobile phase was filtered using a 0.45 µm mixed-cellulose filter membrane and then degassed. The mobile phase was set to flow at a rate of 1 mL/min, and the oven temperature was maintained at 50 °C. For HPLC analysis, all injection volumes were 20 µL. UV detection was performed at a wavelength of 210 nm. The CLR peaks were detected at a retention time of 6.5 min. Solutions of CLR in methanol at drug concentrations ranging from 0.069 to 1.38 mg/mL were used to construct a standard calibration curve (R^2^ = 0.990). Quantitative analysis was performed in triplicate.

### 2.11. Statistical Analysis

All of the experiments were carried out in triplicate. The data was represented as mean ± SD. The unpaired t-test was used to conduct the statistical analysis of the difference in the in vitro release, steady state flux (Jss), permeation (Q) among predetermined intervals among the formulations, and cytotoxicity studies. A *p* value < 0.05 was set as the level of significance.

## 3. Results and Discussion

### 3.1. CLR Saturation Solubility Determination

The solubility of CLR was higher in PBS containing 20% isopropanol than in PBS (pH = 7.4) alone, as summarized in Table 3. Therefore, PBS containing 20% isopropanol was chosen as the receptor media in both the drug release and drug permeation studies to maintain the sink conditions.

### 3.2. Characterization and Assessment of CLR-Loaded Niosomes

#### 3.2.1. Particle Shape and Morphology

**Figure 2:**
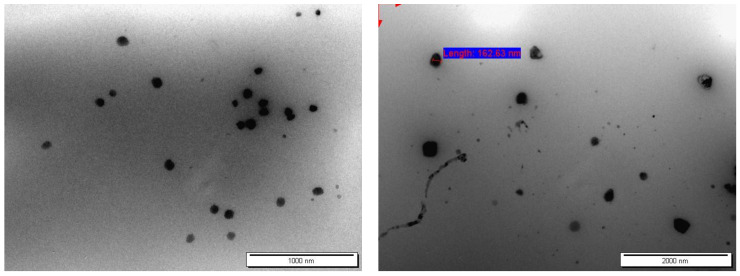
TEM photomicrographs of the selected CLR-loaded niosomal formulation (F1). Particle size of niosome (red arrows).

Figure 2 illustrates the morphology of the niosomal formulation (F1). It was proven that the majority of the produced niosomes had a spherical shape. As illustrated in Figure 2, niosomal formulation (F1) possessed a PS of 162.63 nm, which was relatively close to those obtained by the particle size analyzer of 143.3 ± 24.9 nm, as shown in Table 4. Therefore, the findings of the particle size analyzer in terms of the particle size and uniformity were supported by the TEM analyses. In general, the particles size measured by TEM was usually smaller when using the DLS method, but the particle size measured by TEM could be comparable to the DLS method, as described in previous studies [33,34,35,36].

The PS, ZP, and PDI of the lipid vesicles are summarized in Table 4. The PS values for all the formulations ranged from 109 to 552.1 nm. Nanovesicles with a diameter of 300 nm or less can deliver their contents into deeper skin layers to some extent. On the other hand, particles in the 10–210 nm size range may preferentially penetrate via the transfollicular route [37], indicating that most of the fabricated niosomes could penetrate deeply into hair follicles. Therefore, the niosomes with a PS less than 300 nm were considered to be in the optimum range for use as nanocarriers for transdermal drug delivery. The type of surfactant and the amount of Chol in the formulation affect the size of niosomes. Additionally, the HLB values of surfactants have an impact on the size of niosomes [38]. For samples F1 and F6, in which the ratio of the surfactant: Chol for both formulations was 1:1 and the values of HLB were 9.85 and 15, respectively, the PS values were 143.3 ± 24.9 and 109 ± 11.2 nm, respectively. The niosomes prepared using Span 60 showed significantly (*p* < 0.05) higher particle sizes than the particles prepared using Tween 80. As the HLB value decreased, the more lipophilic surfactant was present, and the PS increased, which was in agreement with literature [39,40]. Formerly, a study by Shehata and Elsewedy [41] reported that Tween 80 formed smaller niosomes than Span 60, with the particle diameters ranging from 343 to 461 nm when the Tween 80 concentration increased from 25 to 100 mol/mL, respectively. However, when the concentration of Span 60 was increased, the vesicular size was considerably increased from 481 nm to 1701 nm. In addition, when a mixture of Tween 80 and Span 60 (1:1) was evaluated, the intermediate particle sizes ranged from 542 to 958 nm [41].

F7 and F8 had same composition of niosomes (Span 60 and Chol with a ratio of 1:0.5; however, they only differed in terms of their drug contents, which were 200 mg and 100 mg, respectively). However, the results in Table 4 show that the increase in the amount of CLR in the hydration media decreased the EE%, where the EE% of F7 and F8 was 12.00 ± 1.00 and 50.00 ± 1.00%, respectively, which was significantly different (*p* < 0.05). This was because the drug-entrapment efficiency of the niosomes was affected by the drug concentration, where the higher the amount of drug that was added to niosomes, the lower the drug-encapsulation efficiency was, as described in [42,43,44]. A high loading concentration of the drug lowered the entrapment efficiency because a high concentration of the drug hindered vesicle formation, as was previously described in [45]. F9, which was composed of 100 mg Tween 80 and 50 mg Chol, had a considerably (*p* < 0.05) larger PS compared to F6, which was composed of a 1:1 Chol: Tween 80 ratio. In addition, F7 and F8 had significantly (*p* < 0.05) larger PSs than those of F4, since these niosomes had lower Chol to surfactant ratios than F4. Our findings may be explained by the fact that the niosomes with lower Chol levels had larger particle sizes. Our findings were in line with those of Akbari et al. [46], who observed that the PS of niosomes significantly dropped from 444.27 ± 4.86 to 383.67 ± 5.03 nm (*p* = 0.0001) as the ratio of Chol: surfactant increased from 0:10 to 5:5. El-far et al. [47] reported that a greater Chol: Tween 80 ratio significantly reduced the particle size of niosomes. This is because higher Chol levels in niosomes results in a greater bilayer hydrophobicity, which raises the surface energy and reduces the particle size [38,48]. Moreover, as the amount of CLR increased from 100 to 200 mg, the vesicular size increased. A study by Miatmoko et al. [42] revealed that the PS of niosomes increased with the addition of ursolic acid, but the encapsulation efficiency decreased when the amount of ursolic acid was increased. To investigate the influence of DCP addition on the niosomal size, we compared the PSs of F3 and F4, where the ratio of Chol:surfactant:drug was 1:1:1, and the HLB value was 4.7 for both formulations. The only difference between the two formulations was the inclusion of DCP in F3. It was found that the addition of DCP had a significant (*p* < 0.05) effect on the PSs of the niosomes, since the PSs of F3 and F4 were 400 and 169 nm, respectively. Our results were in line with those of Sabry et al. [49] who formulated Galangin niosomal vesicles utilizing different non-ionic surfactants (Span 20, Span 40, and Span 60) and Chol at various molar ratios with or without charge-inducing chemicals such as DCP and Stearylamine. They reported that the addition of DCP significantly increased the size of the niosomes.

When examining the stability of vesicles, ZP is a key physical property. Therefore, ZP was employed to foretell the stability of the nanovesicles [50]. The ZP range of the niosomes ranged from −21.59 to −62.87 mV. Stable niosome particles are those with zeta potentials greater than ±30 mV [51]. Thus, these values offer sufficient repulsion between the vesicles to prevent aggregation and generate stable niosomes. Hint et al. [52] stated that negative ZP values can be explained by the free hydroxyl groups found in the molecules of Chol and surfactants and that Chol incorporated into Fosinopril sodium niosomes resulted in negative ZP values in all formulations regardless of the addition of charge inducers. Additionally, Briuglia et al. [53] reported that the incorporation of Chol into vesicular formulations resulted in a negative ZP change, which was in accordance with our results.

The PDI ranged from 0.005 to 0.360, indicating a uniformity of the particles. The PDI results suggested that the samples in this study were monodispersed stable niosomes, which was indicated by the value being less than 0.5, indicating a good particle distribution and a lower aggregation tendency [54].

#### 3.2.2. Encapsulation Efficiency (EE)

The EE% of the prepared CLR-loaded niosomes varied from 12 to 86%. Interestingly, the EE% of the formulations containing Tween 80 was significantly higher than the EE% of the formulations prepared using Span 60. The highest % EE of CLR was found in F1, F6, and F9 (80.06 ± 2.01%, 76.77 ± 9.71%, and 79 ± 9.71%, respectively), compared to the other formulations that had lower HLB values. This was despite the fact that the majority of the available research has indicated that surfactants with an HLB value in the range of 14 to 17 are not suitable for producing niosomes and that ones with an HLB value of 8 provide niosomes with the highest entrapment efficiency [45]. Our findings were in agreement with study conducted by Sailaja and Shreya [55], who stated that a niosomal formulation with a 1:1 Tween-80-to-drug ratio showed the best naproxen-entrapment efficiency, which is a BCS class II drug. In addition, Samira et al. [56] prepared niosomes for the codelivery of hydrophilic and lipophilic anticancer drugs. The study’s findings showed that Tween 60 with a HLB value 14.9 had a higher entrapment efficiency for hydrophobic (curcumin) and hydrophilic (doxorubicin) drugs than Span 60.

Furthermore, Shehata et al. [41] reported that niosomes prepared from Span 60 resulted in a lower EE% than those prepared from Tween 80. This result could be ascribed to the longer unsaturated side chain of the oleate component of Tween 80 compared with Span 60, which may pack the poorly water-soluble drug curcumin more tightly in the niosomal bilayers. On the other hand, Span 60 is a solid with a transition temperature of 53 °C and a saturated stearic fatty acid side chain [41].

#### 3.2.3. In Vitro Release Studies

For poorly water-soluble drugs, adding solubilizers such as Tween-80, ethanol, and isopropanol in the release medium is recommended [57,58,59]. PBS containing 20% isopropanol at pH 7.4 was chosen as the receptor media in both the drug-release and drug-permeation studies to achieve the required sink conditions. This was because sink conditions are attained when the equilibrium solubility of drugs in the dissolving medium is at least three times the volume required for drug saturation [59]. In addition, these tests were carried out using PBS solution containing 20% isopropanol (pH 7.4) at 37 ± 0.5 °C and 600 rpm to simulate physiological conditions, as has been previously described in [60]. Additionally, to preserve the sink conditions, sampling was conducted at specific time intervals, and after each sampling, a fresh buffer solution was supplied to the release medium [61].

**Figure 3:**
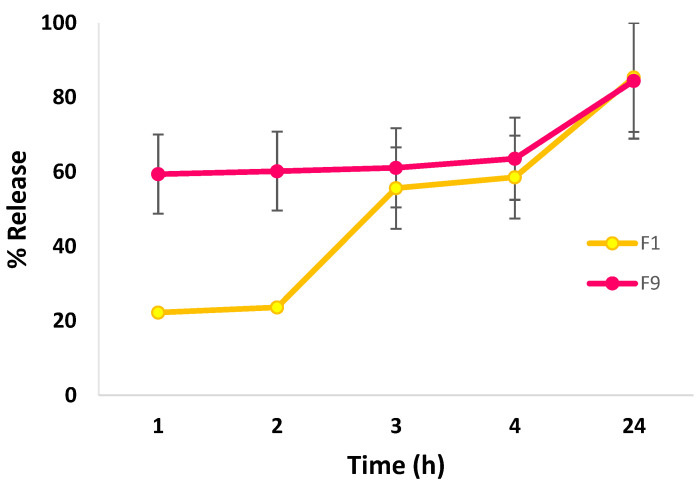
In vitro release studies of CLR from F1 and F9. Data are presented as mean ± SD (*n* = 3).

F1 and F9 were chosen for the in vitro release studies as they had the greatest EE% and a ZP value above −30 mV compared with F6. Figure 3 represents the in vitro release profiles of the CLR-loaded niosomes from F1 and F9. After the first hour, the release results from the F1 and F9 formulations were 22.22 ± 0.59 and 59.39 ± 10.63%, respectively. This difference was considered to be very significant (*p* < 0.005). This result could be due to the variation in the Chol: surfactant ratio between the two formulations, where F1 contained a 1:1 ration and F9 contained a 0.5:1 ratio. Generally, the release rate is lowered as the Chol content is raised, as has been shown in different studies, such as those involving simvastatin [62] and diclofenac sodium [46]. Akbari et al. [46] and Witika et al. [63] reported that the vesicle rigidity will increase and the drug release rate will slow down due to Chol increasing the lipid state bilayer order. However, Yaghoobian et al. [64] reported that the release rate of repaglinide, which is a BCS class II drug, was the slowest in vesicles composed of Span 60. This outcome was attributed to the surfactant’s lipophilic character (HLB = 4.7), which leads to greater drug–lipid chain interactions in the lipid layers of niosomes. The repaglinide release rate from niosomes improved from 54.4 to 82.6% as the HLB of the surfactants increased from 4.70 to 16.9. Interestingly, after 24 h, F1 and F9 showed a comparable release percentage of 85.40 ± 14.64% and 84.47 ± 15.53%, respectively, which was considered insignificant (*p* > 0.05). Our findings corroborated with Taymouri and Varshosaz [65], who found that the overall amount of carvedilol (BCS class II) released from various types of surfactants did not change significantly despite the difference in the HLB values of the surfactants.

#### 3.2.4. Release Kinetics Modeling

**Figure 4:**
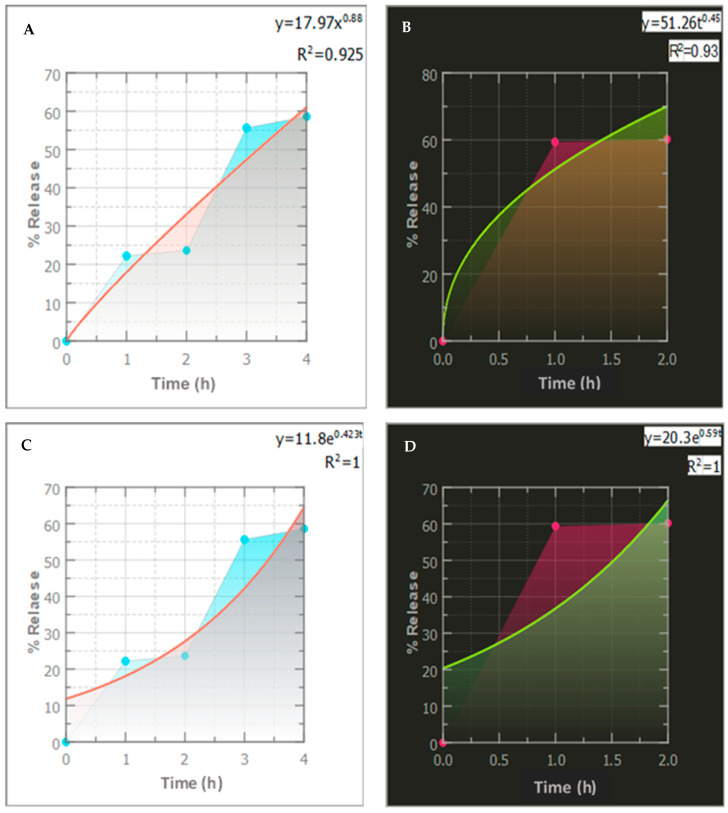
Different kinetic models and their parameters for the release pattern of CLR from F1 and F9 niosomal formulations: Korsmeyer–Peppas kinetic model for (**A**) F1 and (**B**) F9. First-order kinetic model for (**C**) F1 and (**D**) F9.

The mathematical kinetic models were examined to study the kinetics of CLR release from the niosomes. The correlation coefficients (R^2^) and release exponents (*n*) of the kinetic models are displayed in Figure 4. The ideal kinetic model for CLR release for each formulation was determined by the model that exhibited the highest regression coefficient (R^2^) value (close to 1) [66]. As a result, F1 and F9 obeyed a first-order kinetic model, which stated that the drug release rate was proportional to the residual drug concentration in the system. Similar findings have been reported in previous studies [67,68]. For instance, Kulkarni et al. [67] reported that a first-order model was determined to be the most appropriate model from the investigation of the release kinetics of hyaluronic-acid-loaded niosomes, as evidenced by an R^2^ value of 0.99. In another study, Saafan et al. [68] showed that first-order kinetics provided the most accurate representation of drug release for a chloroquine–niosomal formulation.

According to the Korsmeyer–Peppas equation, the equivalent graph of the log cumulative percentage drug release vs time is represented in Table 5, which had a good linearity R^2^ of 0.925 and 0.93, while the release exponent (*n*) was 0.88 and 0.45 for F1 and F9, respectively (Figure 4). F1 exhibited anomalous transport kinetics (*n* = 0.53–0.63), implying diffusion and degradation mechanisms, whereas F9 exhibited a value of *n* = 0.45, implying a Fickian diffusion (Case I) that was representative of first-order kinetics where diffusion was the main release mechanism.

### 3.3. Antimicrobial Activity of CLR-Loaded Niosomes

**Figure 5:**
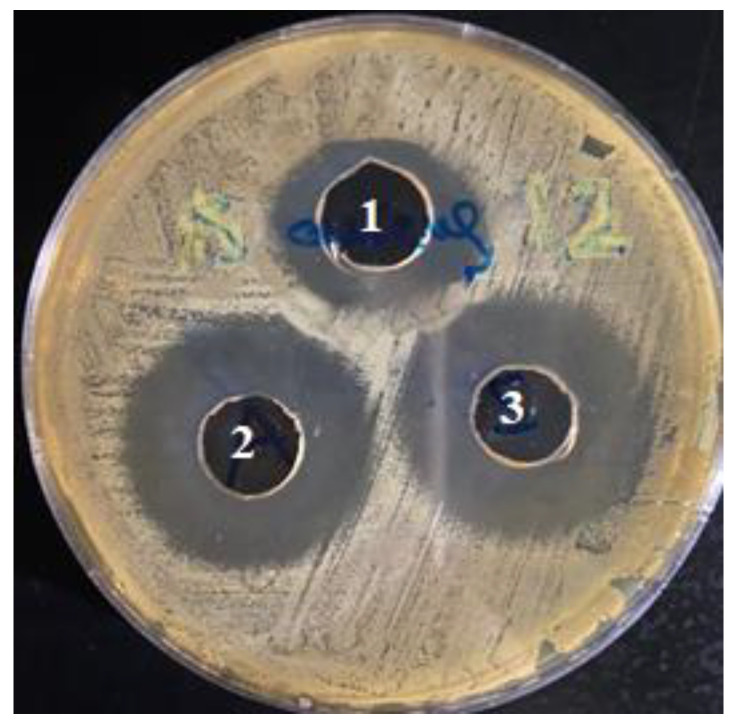
The zones of inhibition of S. aureus using the niosomal formula F9. Note: well no.1: negative control (50% DMSO: PBS); well no.2: positive control (CLR 12 μg/100 μL); well no.3: test item (niosomal formulation F9) (CLR 12 μg/100 μL).

The antimicrobial activity of the CLR-loaded niosomes (F9) was assessed by measuring the zones of inhibition of Gram-positive bacteria (*S. aureus*, ATCC 6538) and Gram-negative bacteria (*E. coli*, ATCC 8739) after 24 h in a bacterial culture. Table 5 illustrates the results and zones of inhibition. Both the CLR and F9 inhibited S. aureus more than *E-coli*. As can be clearly observed from Figure 5, the CLR encapsulation in the niosomes showed antibacterial action against *S. aureus,* which was similar to a free drug. Based on these findings, the niosomal formulation maintained the antimicrobial activity of the CLR without affecting the effective concentration. This result agreed with the study in [32], which showed that niosomes containing azithromycin exhibited an antibacterial activity against *S. aureus* and *S. epidermidis* that was comparable to that of free azithromycin.

### 3.4. Fabrication of CLR-Loaded Niosomal Patches (CLR–NP)

In the current study, matrix-type transdermal patches were developed and evaluated. Cellulose derivatives such as HPMC and Na-CMC were used to prepare these patches. PEG 400 was used as a plasticizer, which is frequently employed in a concentration between 5 and 20% (*w*/*w*). PEG 400 acts as a penetration enhancer, increasing skin permeability and enhancing the diffusivity and solubility of drugs through the skin [26]. The selected niosomal formulation (F9) was incorporated into patches at different levels (5–20 *w*/*w*%). The CLR–NPs were developed using HPMC, Na-CMC, PEG 400, and a CLR–niosomal dispersion (F9). The findings demonstrated that only two of the formulations offered flexible and uniform patches after being cast into molds (P3 and P4). These were prepared using a mixture of 45% HPMC, 35% Na-CMC, 15% PEG400, 5% CLR–niosomal dispersion (P3), and 40% HPMC, 30% Na-CMC, 15%PEG-400, and 15% CLR–niosomal dispersion (P4), as illustrated in Table 3. Zaid Alkilani et al. [26], in their previous study, used various patch-forming polymers, including Na-CMC, polyvinyl alcohol, polyvinyl pyrrolidone, and HPMC, as well as two plasticizers (PEG 400 and isopropanol), and they stated that the optimal patches were prepared from Na-CMC and HPMC aqueous blends.

### 3.5. Evaluation of CLR-Loaded Niosomal Patches

**Figure 6:**
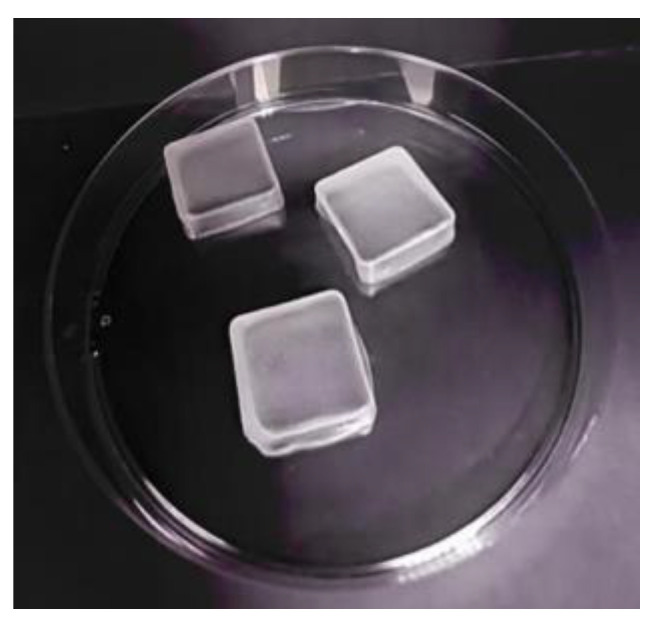
Appearance of matrix-type patches containing CLR niosomes (P4) under digital camera.

The thickness, weight uniformity, moisture content, drug content, and pH of the samples are presented in Table 6. The weights were 415.4 ± 0.6 mg and 421.6 ± 0.7 mg for P3 and P4, respectively. The thickness of P3 was 0.414 ± 0.014 mm, while that of P4 was 0.425 ± 0.020 mm. According to the HPLC analysis, the CLR contents of the P3 and P4 patches were 2.12 ± 0.12 mg and 6.37 ± 0.35 mg, respectively (P3 had a 5% drug content and P4 had a 15% drug content), accounting for 96.58 ± 5.38% and 97.57 ± 5.43%. This demonstrated the uniform drug distribution in P3 and P4. The moisture content was found to be 16.82 ± 0.11% and 9.89 ± 0.10% for P3 and P4, respectively, with a significant difference (*p* < 0.001) between P3 and P4. It is also worth noting that the required moisture content for transdermal patches is less than 10% [69]. P3 was therefore considerably unstable and vulnerable to microbial contamination. This was due to the greater levels of Na-CMC in the formulations, which resulted in an increase in the moisture content. Our findings were in agreement with Latif et al. [70] who revealed that Na-CMC is a hygroscopic polymer that can absorb and retain water in transdermal patches, resulting in a greater moisture content than for the HPMC polymer. Therefore, P4 was chosen for further characterization. Additionally, the surface pH was maintained at 4.84 and 4.74, which was compatible with the skin’s pH of 4–6 [71]. After three days, the physical and organoleptic features of the patches, such as their color, flexibility, smoothness, and brittleness, were investigated. The prepared patches had a uniform matrix, a smooth surface with no visible cracks, and were elastic, as shown in Figure 6.

### 3.6. ATR–FTIR

**Figure 7:**
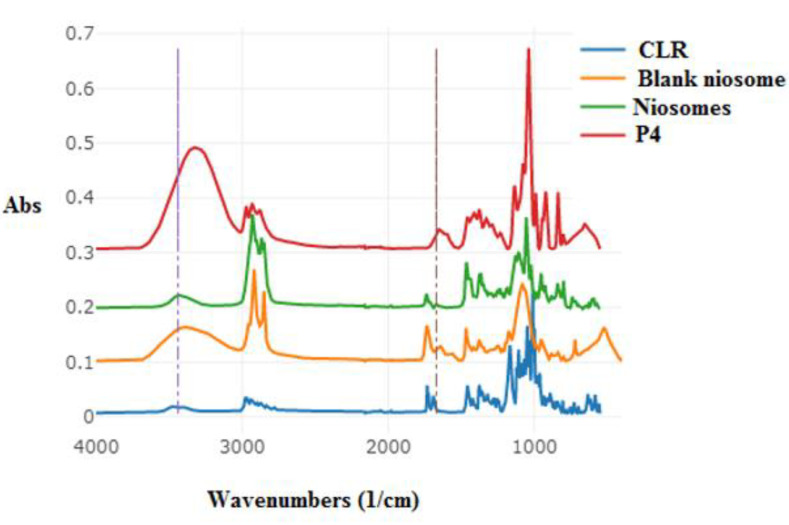
ATR–FTIR spectra of CLR, blank niosomes, niosomes, and niosomal patch (P4).

The compatibility of CLR with other the components employed in developing the CLR-loaded niosomal patch P4 was investigated (Figure 7). In the spectrum of the drug-loaded niosomes, notable peaks were observed at 3370, 2920, and 1738 cm^−1^, which corresponded to OH stretching, a carbonyl dimer, and C=O stretching, respectively [72]. Similar to what was seen in the spectrum of the blank niosomes, significant shifts were observed in terms of C=O stretching, which were indicative of interactions mediating niosome formation [73]. The shift seen in the OH-stretching peak suggested that the likely mechanism by which CLR was incorporated into the niosome was hydrogen bonding [74].

In the prepared patches of polymeric blends, the principle CLR bands were recognized. The FTIR analysis of the CLR-loaded niosomal patch and the physical mixture revealed minor peak shifting and smoothening, showing a physical interaction between the CLR and the components that made up the niosomal patch (P4) [75].

### 3.7. Rheological Studies

**Figure 8:**
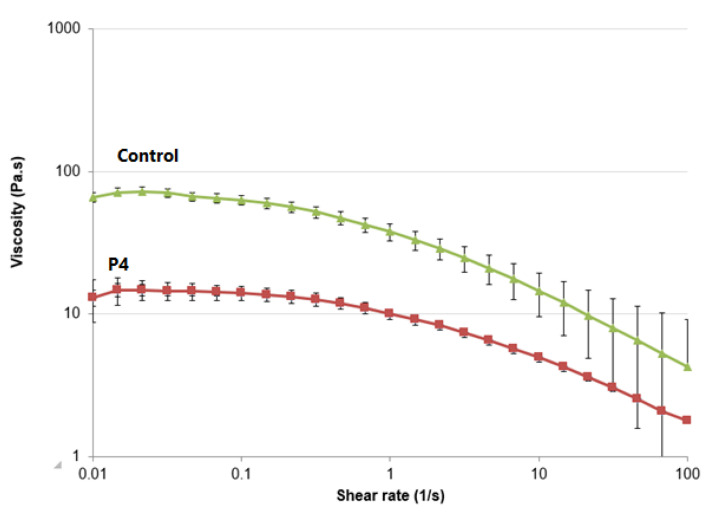
The effect of shear rate on the viscosity of CLR-loaded niosomal patches (P4) and conventional CLR patches (control).

The viscosity of the polymeric formulations, used in patch preparation, was examined to estimate the flow of CLR release from patches [76]. Both the CLR-loaded conventional patches (control) and the CLR-loaded niosomal patches (P4) displayed a pseudo-plastic (thinning) flow with high viscosity at low shear rates and decreasing viscosity at higher shear rates [77], as shown in Figure 8. This confirms that the shear-thinning characteristic of CLR-loaded niosomal patches was maintained by the incorporation of niosomes. Similarly, in investigations conducted by El Ridy et al. [78], lornoxicam niosomal gel and lornoxicam loaded gel were developed. Shear-thinning (pseudo-plastic) behavior was observed in the prepared gels. This shear-thinning action was attributed to the instant arrangement of the colloidal network structure into layers that can flow more freely over each other in the direction of shear, and therefore the viscosity decreases as the shear rate increases [78].

Notwithstanding, P4 revealed lower viscosity than its corresponding conventional patches that contain free-CLR (control), as evidenced by the flow curves. Adding niosomes to the aqueous polymeric gels may be responsible for P4 decreased viscosity. Moreover, Manosroi et al. [54] have also noted a decrease in the viscosity of Carbopol^®^ hydrogels after incorporating niosomes. This decreased viscosity was attributed to the presence of surfactants in the niosomes that may have promoted a weakening of the inter-polymer bonds [79].

### 3.8. In Vitro Permeation Study

**Figure 9:**
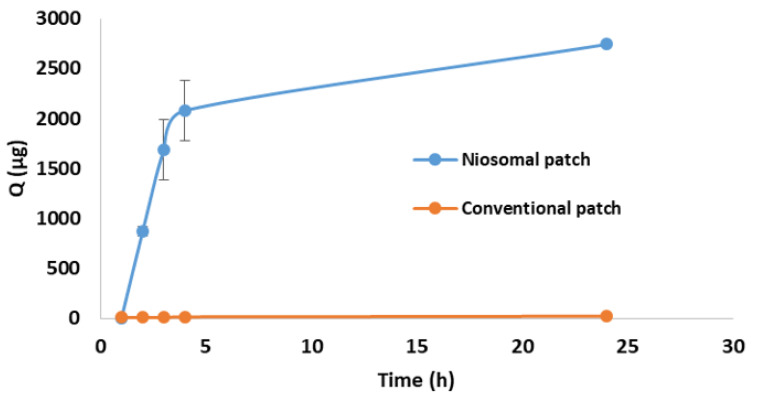
Cumulative amount of CLR that permeated from niosomal and conventional patches across Strat-M^®^ membrane for 24 h. Data are presented as mean ± SD (*n* = 3).

An in vitro permeation study on a Strat-M^®^ membrane was conducted to assess the permeation pattern of the CLR from P4. Figure 9 shows the in vitro permeation profiles of the conventional patch, which was used as a control, and CLR–niosome-loaded patch (P4). The data presented in Table 7 indicate that the flux (Jss) of the niosomal patch (P4) was significantly higher than that of the conventional patch by more than 200 times. When comparing the CLR release data from the niosomal and conventional patches, the niosomal patch had a significantly (*p* < 0.05) higher permeability coefficient of CLR than the conventional patch, with values of (102.1 ± 0.6) × 10^−3^ cm/h and (0.5 ± 0.0) × 10^−3^ cm/h, respectively. The permeation profile of P4 revealed a progressive rise in the CLR concentration over 24 h. The cumulative amount of 2742 µg of CLR that permeated the membrane after 24 h from the P4 sample was compared to the cumulative amount of CLR that permeated from the conventional patch (20 µg) after 24 h. Thus, the difference among them was significant (*p* < 0.05).

Our findings were consistent with previous research that found an increase in drug permeation after encapsulating the studied drug in vesicular nanocarriers. The addition of nonionic surfactants in niosomal formulations serves as a permeation enhancer, potentially improving drug penetration through skin layers [2,9,11,45]. Furthermore, by enhancing skin hydration, nanosized niosomes can also disrupt the tightly packed cellular structure of the SC [80]. Fahmy et al. [80] investigated the effect of niosomes as a nanocarrier on the skin permeation of ozonated olive oil. At 24 h, the skin permeation of the free ozonated olive oil was 17.24 ± 2.06%, while the skin permeation of ozonated-olive-oil niosomes was 53.44 ± 6.41% after 24 h. This was ascribed to the presence of Span 60 and Tween 60, which served as permeation enhancers [80]. In addition, Hashemi et al. [81] reported that the amount of Chol in niosomes and the HLB value appeared to significantly influence how venlafaxine HCl permeated through the skin. The highest level of venlafaxine HCl measured in the receptor compartment for conventional gel was significantly lower (*p* < 0.05) than that for niosomal gel [81]. Chol has been shown to be able to alter membrane fluidity and phase behavior, which may increase the absorption of drugs through the skin [81].

Furthermore, El-Ridy et al. [78] revealed that the penetration of lornoxicam encapsulated in niosomes was superior to that of conventional lornoxicam gel. The improved skin absorption of lornoxicam from the niosomal gel was attributed to the presence of nonionic surfactants, which acted as permeation enhancers, and to the niosomal vesicles in the nanometer size range, which exhibited superior occlusive properties compared to plain aqueous gel. These properties improved skin moisture and skin penetration. Moreover, the higher viscosity of the conventional gel signified a decrease in the concentration of the permeating drug (Figure 8). Previous studies by Hamed et al. [82,83] showed that viscous gels exhibited slower release profiles due to the high complexity of the gel network resulting in a long diffusion pathway. Numerous studies have demonstrated that niosomal gels have higher retention and penetration rates through various skin layers than their corresponding free form in solutions or conventional gels, such as for acyclovir [84], etodolac [85], resveratrol [86], and pregabalin [87]. These findings were justified by the lower molecular mobility and diffusion associated with the increased viscosity of the conventional gels [79]. Thus, by encapsulating CLR niosomes into transdermal patches, the CLR transdermal delivery was significantly enhanced in a controlled manner.

### 3.9. Cytotoxicity

**Figure 10:**
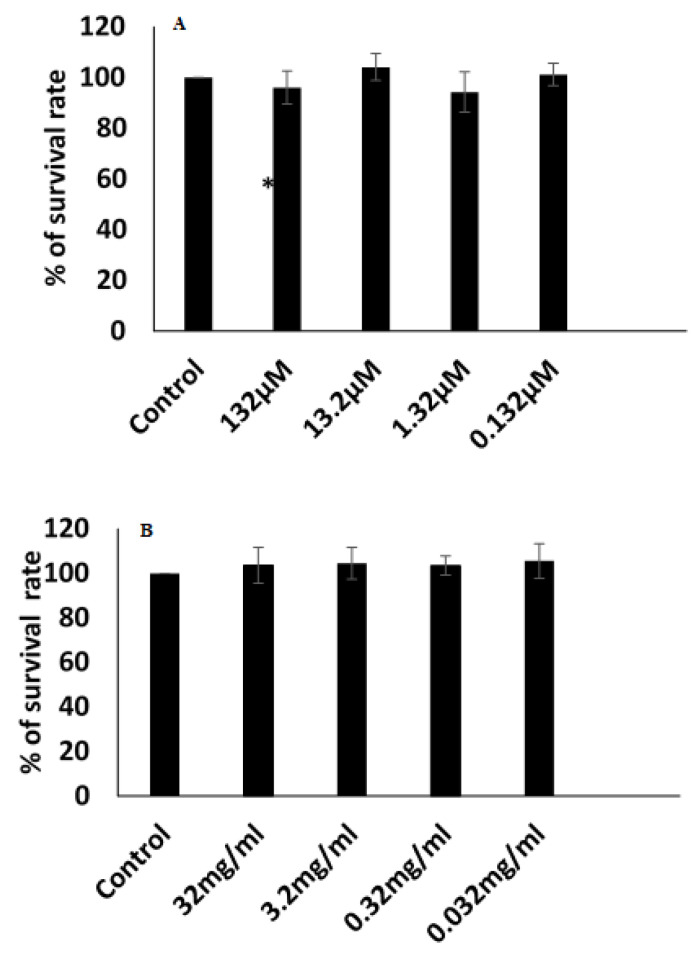
Cytotoxicity of (**A**) CLR niosomal patch (P4), (**B**) blank transdermal patch. No cytotoxic effect on MCF-7 cells was observed. * *p* < 0.01 being considered significant.

The repurposing of antibiotics as anticancer agents has led to the discovery of new anti-cancer drugs [88]. Therefore, we evaluated the cytotoxic effect of a CLR transdermal patch (P4) on MCF-7 cells with an MTT assay. Our results showed no cytotoxic effects, with values of 0.132 μM to 132 μM (Figure 10A). We also examined the cytotoxicity of a blank transdermal patch (without CLR at concentrations of 0.032–32 mg/mL). Again, no cytotoxicity was observed with the blank patch (Figure 10B). Although we could not detect any cytotoxic effect of CLR on breast cancer cells, our results confirmed the safety of the ingredients used to develop the transdermal patch to enhance the anti-microbial effect of CLR.

## 4. Conclusions

CLR-loaded niosomal nanovesicles were successfully prepared and incorporated into a skin patch for transdermal administration. Interestingly, the encapsulation efficiency of the formulations containing Tween 80 was significantly higher than that of the formulations prepared using Span 60. The optimal Chol: surfactant ratio for drug release was found to be 0.5:1. In addition, the encapsulation of CLR into niosomal nanovesicles did not reduce the antibacterial activity of the CLR. The shear-thinning characteristics of the CLR-loaded niosomal patches were maintained by the incorporation of niosomes. The niosomal patches had a significantly higher permeability coefficient of CLR than the conventional patch, and they significantly enhanced the transdermal delivery of CLR. Proceeding from the aforementioned findings, it can be concluded that niosome-based patches that mediate TDD are a potential strategy for avoiding the bitterness of the oral liquid dosage form of CLR, lowering GIT-related side effects, and extending CLR’s presence in the systemic circulation, thus, allowing for less frequent dosing. In conclusion, niosome-based transdermal patches may be a promising method for the TDD of class II drugs and drugs experiencing GIT-related side effects.

## Figures and Tables

**Table 1:** Composition of CLR-loaded niosomal formulations.

Formulation Code	Span 60	Span 40	Span 80	Tween 80	DCP	Cholesterol	CLR	HLB
	(mg)	(mg)	(mg)	(mg)	(mg)	(mg)	(mg)	Value
F1	50	-	-	50	-	100	100	9.85
F2	-	100	-	-	5	100	200	6.7
F3	100	-	-	-	5	100	200	4.7
F4	100	-	-	-	-	100	100	4.7
F5	-	-	100	-	5	100	200	4.3
F6	-	-	-	100	-	100	100	15
F7	200	-	-	-	-	100	200	6.7
F8	200	-	-	-	-	100	100	6.7
F9	-	-	-	100	-	50	100	15

**Table 2:** Composition of CLR–NP using different polymers.

Code	HPMC	Na-CMC	PEG-400	CLR-Niosomal Dispersion
	*w*/*w* (%)	*w*/*w* (%)	*w*/*w* (%)	*w*/*w* (%)
P1	60%	-	20%	20%
P2	-	60%	20%	20%
P3	45%	35%	15%	5%
P4	40%	30%	15%	15%

**Table 3:** CLR saturation solubility results in PBS and PBS containing 20% isopropanol (mean ± SD, *n* = 3).

Media	Solubility (mg/mL)
PBS	0.12 ± 0.01
PBS containing 20% isopropanol	0.37 ± 0.17

**Table 4:** PS, ZP, PDI, and EE% values for the prepared formulations of CLR-loaded niosomes (mean ± SD, *n* = 3).

Code	PS (nm)	PDI	ZP (mV)	% EE
F1	143.30 ± 24.90	0.15 ± 0.08	−40.86 ± 1.50	86.00 ± 2.00
F2	300.00 ± 5.60	0.34 ± 0.00	−50.10 ± 0.42	15.00 ± 2.00
F3	400.00 ± 3.60	0.36 ± 0.00	−51.82 ± 0.22	20.00 ± 4.00
F4	169.20 ± 11.90	0.18 ± 0.04	−53.83 ± 0.53	52.00 ± 2.00
F5	425.70 ± 10.80	0.34 ± 0.00	−51.04 ± 0.17	13.00 ± 1.00
F6	109.00 ± 11.20	0.27 ± 0.04	−21.59 ± 2.01	82.00 ± 7.00
F7	437.00 ± 72.30	0.04 ± 0.04	−28.42 ± 1.91	12.00 ± 1.00
F8	552.10 ± 17.50	0.00 ± 0.00	−59.08 ± 2.15	50.00 ± 1.00
F9	295.20 ± 71.40	0.00 ± 0.00	−36.22 ± 1.77	79.00 ± 10.00

**Table 5:** Zones of inhibition (mm) of the tested niosomal formulation (F9) against *S. aureus* and *E-coli.* Well no.1: negative control (NC) (DMSO + PBS); well no. 2: positive control (PC) with a CLR of 12 μg/100 μL and a CLR of 30 μg/100 μL for *S. aureus* and *E-coli*, respectively. Well no. 3: F9 (test sample) with a CLR of 12 μg/100 μL and a CLR of 30 μg/100 μL for *S. aureus* and *E. coli*, respectively.

	Zones of Inhibition (mm)		
Bacterial Strain	Well (1)	Well (2)	Well (3)
*Staphylococcus aureus*	2.4	3.2	3.2
*Escherichia coli*	1.3	1.9	2.0

**Table 6:** Physicochemical properties of CLR niosomal patches P3 and P4. (Mean ± SD, *n* = 4).

CLR–NP	Thickness (mm)	Weight Uniformity (mg)	Moisture Content (%)	Drug Content (%)	pH
P3	0.42 ± 0.02	415.40 ± 0.60	16.82 ± 0.11	96.58 ± 5.38	4.84 ± 0.01
P4	0.41 ± 0.01	421.60 ± 0.70	9.89 ± 0.10	97.57 ± 5.43	4.74 ± 0.01

**Table 7:** Drug penetration parameters from niosomal and conventional patches at 32 °C. Data are presented as mean ± SD, (*n* = 3).

Formulations	Jss (µg/cm²/h)	*p* × 10^−3^ (cm/h)
Niosomal patch (P4)	380.2 ± 2.3	102.1 ± 0.6
Conventional patch (control)	1.7 ± 0.0	0.5 ± 0.0

## Data Availability

Not applicable.
